# Ghrelin delays premature aging in Hutchinson‐Gilford progeria syndrome

**DOI:** 10.1111/acel.13983

**Published:** 2023-10-19

**Authors:** Marisa Ferreira‐Marques, André Carvalho, Ana Catarina Franco, Ana Leal, Mariana Botelho, Sara Carmo‐Silva, Rodolfo Águas, Luísa Cortes, Vasco Lucas, Ana Carolina Real, Carlos López‐Otín, Xavier Nissan, Luís Pereira de Almeida, Cláudia Cavadas, Célia A. Aveleira

**Affiliations:** ^1^ CNC – Center for Neuroscience and Cell Biology University of Coimbra Coimbra Portugal; ^2^ CIBB – Center for Innovative Biomedicine and Biotechnology University of Coimbra Coimbra Portugal; ^3^ Faculty of Pharmacy University of Coimbra Coimbra Portugal; ^4^ Departamento de Bioquímica y Biología Molecular, Facultad de Medicina, Instituto Universitario de Oncología Universidad de Oviedo Oviedo Spain; ^5^ CECS, I‐Stem Corbeil‐Essonnes France; ^6^ INSERM U861, I‐Stem Corbeil‐Essonnes France; ^7^ UEVE U861, I‐Stem Corbeil‐Essonnes France; ^8^ MIA‐Portugal – Multidisciplinar Institute of Ageing University of Coimbra Coimbra Portugal

**Keywords:** autophagy, ghrelin, human aging, progeria, senescence

## Abstract

Hutchinson‐Gilford progeria syndrome (HGPS) is a rare and fatal genetic condition that arises from a single nucleotide alteration in the *LMNA* gene, leading to the production of a defective lamin A protein known as progerin. The accumulation of progerin accelerates the onset of a dramatic premature aging phenotype in children with HGPS, characterized by low body weight, lipodystrophy, metabolic dysfunction, skin, and musculoskeletal age‐related dysfunctions. In most cases, these children die of age‐related cardiovascular dysfunction by their early teenage years. The absence of effective treatments for HGPS underscores the critical need to explore novel safe therapeutic strategies. In this study, we show that treatment with the hormone ghrelin increases autophagy, decreases progerin levels, and alleviates other cellular hallmarks of premature aging in human HGPS fibroblasts. Additionally, using a HGPS mouse model (*Lmna* ^G609G/G609G^ mice), we demonstrate that ghrelin administration effectively rescues molecular and histopathological progeroid features, prevents progressive weight loss in later stages, reverses the lipodystrophic phenotype, and extends lifespan of these short‐lived mice. Therefore, our findings uncover the potential of modulating ghrelin signaling offers new treatment targets and translational approaches that may improve outcomes and enhance the quality of life for patients with HGPS and other age‐related pathologies.

## Graphical abstract

Ghrelin, by promoting progerin clearance, delays cellular aging markers in human Hutchinson‐Gilford progeria syndrome (HGPS) fibroblasts. Ghrelin reverts lipodystrophy and improves metabolic profile dysfunction phenotype in progeria mouse model. Ghrelin enhances lifespan and healthspan in progeria mouse model.

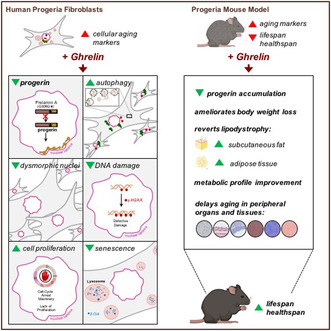

**Abbreviations**
BAT: brown adipose tissue
ChQ: chloroquine
GHS‐R1a: growth hormone secretagogue receptor
HGPS: Hutchinson‐Gilford progeria syndrome
KRT‐1: keratin‐1
LC3: microtubule‐associated protein 1A/1B‐light chain 3
mTOR: mammalian target of rapamycin
SA‐β‐Gal: senescence‐associated‐β‐galactosidase
SQSTM1: sequestosome 1
VSMCs: vascular smooth muscle cells
WAT: white adipose tissue
α‐SMA: alpha‐smooth muscle actin

## 1 INTRODUCTION

Hutchinson‐Gilford progeria syndrome (HGPS; OMIM#176670) is a rare and fatal genetic condition characterized by premature and accelerated aging, with an incidence of approximately one in 4–8 million live births (Gordon et al., 2014; Merideth et al., 2008; Ullrich & Gordon, 2015). Children with HGPS exhibit low body weight, lipodystrophy, abnormalities in the skin, musculoskeletal, and cardiovascular systems, and typically die from myocardial infarction or stroke at a median age of 14.6 years (Gordon et al., 2014; Merideth et al., 2008; Ullrich & Gordon, 2015). In the classical form of HGPS, a specific mutation (c.1824C > T,p.G608G) causes a single C‐to‐T transition point mutation at position 1824 of the *LMNA* gene. This mutation leads to the activation of a previously hidden splice site, resulting in the loss of the terminal 150 nucleotides of exon 11 (De Sandre‐Giovannoli et al., 2003; Eriksson et al., 2003). The consequence of this deletion in the C‐terminal domain is the formation of a truncated form of prelamin A, named progerin. Progerin does not undergo the normal posttranslational processing retaining a toxic farnesyl modification. Permanent farnesylation causes progerin accumulation in the inner nuclear membrane, which is at least partly responsible for the HGPS phenotype (Gonzalo et al., 2017).

Ghrelin is a 28‐aa Ser3 acylated peptide, and its functionally relevant endogenous receptor is the growth hormone secretagogue receptor (GHS‐R1a) ghrelin levels and/or its signaling are decreased in elderly individuals, which may be associated with age‐related alterations (Akamizu et al., 2006; Rigamonti et al., 2002; Yin & Zhang, 2016). In addition, our group has previously showed that ghrelin stimulates autophagy, a proteostasis mechanism impaired in natural aging and HGPS (Ferreira‐Marques et al., 2016, 2021). Also, we and others showed that rapamycin, a known‐autophagy stimulator, decreases progerin and delay aging progression in HGPS cells (Aveleira et al., 2020; Cao et al., 2011); therefore, we hypothesized that ghrelin may act on similar pathways, exerting its beneficial effects through stimulation of autophagy and progerin clearance, rescuing the senescent phenotype of HGPS cells, ultimately delaying or blocking the aging phenotype of HGPS and potentially increasing lifespan. Here, using fibroblasts from HGPS patients and an animal model of HGPS, the *Lmna* ^*G609G/G609G*^ mice, we report that ghrelin delays premature aging of HGPS, and consequently improves longevity.

## 2 RESULTS

### 2.1 Ghrelin increases autophagy and enhances progerin clearance in HGPS cells

**FIGURE 1:**
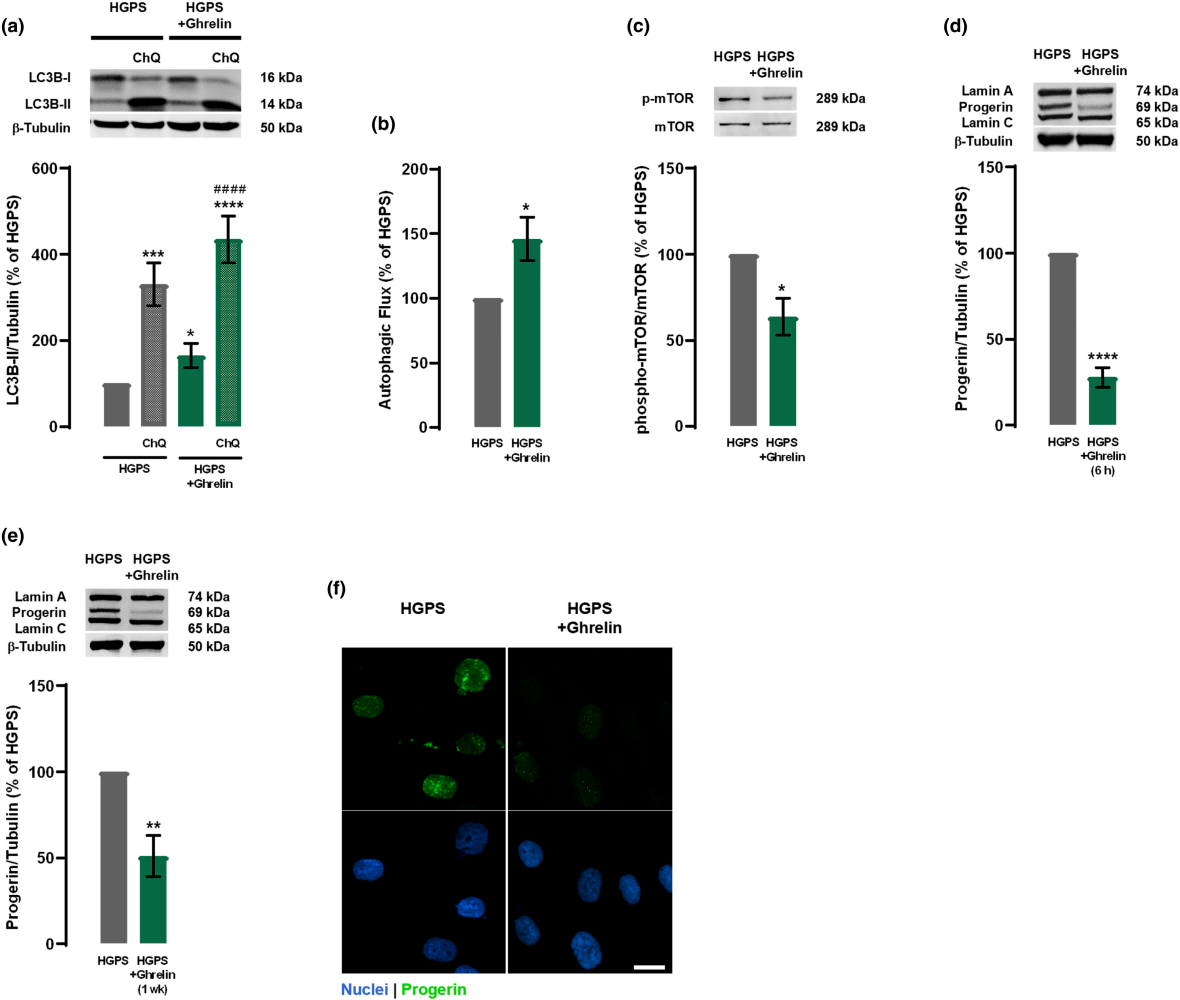
Ghrelin enhances autophagy and progerin clearance in HGPS fibroblasts. (a–f) HGPS fibroblasts were exposed to ghrelin (1 nM) for 6 h (HGPS + Ghrelin) in the presence or absence of chloroquine (ChQ, 100 μM), a lysosomal degradation inhibitor (a–d), or for 1 week, treated every other day (e and f). HGPS‐untreated cells were used as control (HGPS). Whole‐cell extracts were assayed for LC3B (a) (*N* = 4), p‐mTOR/mTOR (c) (*N* = 3), lamin A/progerin/lamin C (d and e) (*N* = 5) and β‐tubulin (loading control) immunoreactivity through western blotting analysis. Representative western blots for each protein are presented above each respective graph. Autophagic flux analysis in HGPS cells is shown (b) (*N* = 4). Autophagic flux was determined in the presence of the lysosomal inhibitor chloroquine and expressed as “Autophagic flux” calculated by subtracting the densitometric value of LC3B‐II‐ChQ from those corresponding LC3B‐II + ChQ values. (f) Ghrelin decreased progerin immunoreactivity. Cells were immunolabeled for progerin (top panels, green) and nuclei were stained with Hoechst 33342 (bottom panels, blue). Images are representative of three independent experiments. Scale bar, 10 μm. Data are expressed as the mean ± SEM of at least three independent experiments and are expressed as a percentage of HGPS. *p < 0.05, **p < 0.01, ***p < 0.001, and ****p < 0.0001 significantly different compared to HGPS; ^####^ *p* < 0.0001, significantly different compared to HGPS + Ghrelin, as determined by analysis of variance, followed by Tukey's multiple comparison test, or Student's *t* test. HGPS, Hutchinson‐Gilford progeria syndrome.

One of the hallmarks of cellular aging in HGPS is loss of proteostasis and autophagy impairment, which could be one of the major promoters of progerin accumulation in HGPS cells (Gabriel et al., 2015). Using rodent neurons, we previously demonstrated that ghrelin activates autophagy (Ferreira‐Marques et al., 2016, 2021). Therefore, we hypothesized that ghrelin could promote progerin clearance through autophagy induction in fibroblasts from HGPS patients. Ghrelin treatment (6 h) increased LC3B‐II levels in HGPS cells (Figure 1a). To investigate if this increase in LC3B‐II was due to an inhibited autophagosome degradation rather than autophagosome formation, we evaluated the endogenous autophagic flux in the absence and presence of chloroquine, an inhibitor of autophagic degradation (Klionsky et al., 2016) (Figure 1b), supporting that ghrelin stimulates autophagy in HGPS cells. In addition to LC3B‐II, ghrelin also increased the autophagic degradation of SQSTM1, a well‐known autophagic substrate (Figure S1a,b). Altogether, these findings demonstrate that ghrelin enhances autophagic clearance in HGPS‐derived cells. Ghrelin also significantly decreased phospho‐mTOR protein levels (Figure 1c), indicating that the observed autophagy stimulation occurs by inhibiting mTOR in HGPS cells. This finding is consistent with our previous observations that rapamycin, positive control for autophagy induction, increased LC3B‐II protein levels, a marker of autophagosome formation, and boosted autophagic flux through inhibiting mTOR (Aveleira et al., 2020). Concomitant with this upregulation in autophagy, ghrelin‐treated HGPS cells showed lower progerin protein levels (Figure 1d) compared to untreated HGPS cells. This effect was observed not only after 6 h treatment, but also when HGPS cells were exposed to ghrelin for 1 week, treated every other day (Figure 1e), suggesting that ghrelin leads to sustained progerin clearance. Rapamycin also decreased progerin levels (Aveleira et al., 2020; Cao et al., 2011), further supporting a role for autophagy in progerin clearance. Additionally, ghrelin‐treated HGPS cells exhibited very low, or even absent, progerin immunoreactivity (Figure 1f). Although progerin protein levels were decreased upon ghrelin treatment, its mRNA expression was not affected (Figure S2a,b). These results suggest that ghrelin promotes progerin clearance in HGPS cells.

### 2.2 Ghrelin rescues aberrant nuclear morphology and decreases DNA damage in HGPS cells

**FIGURE 2:**
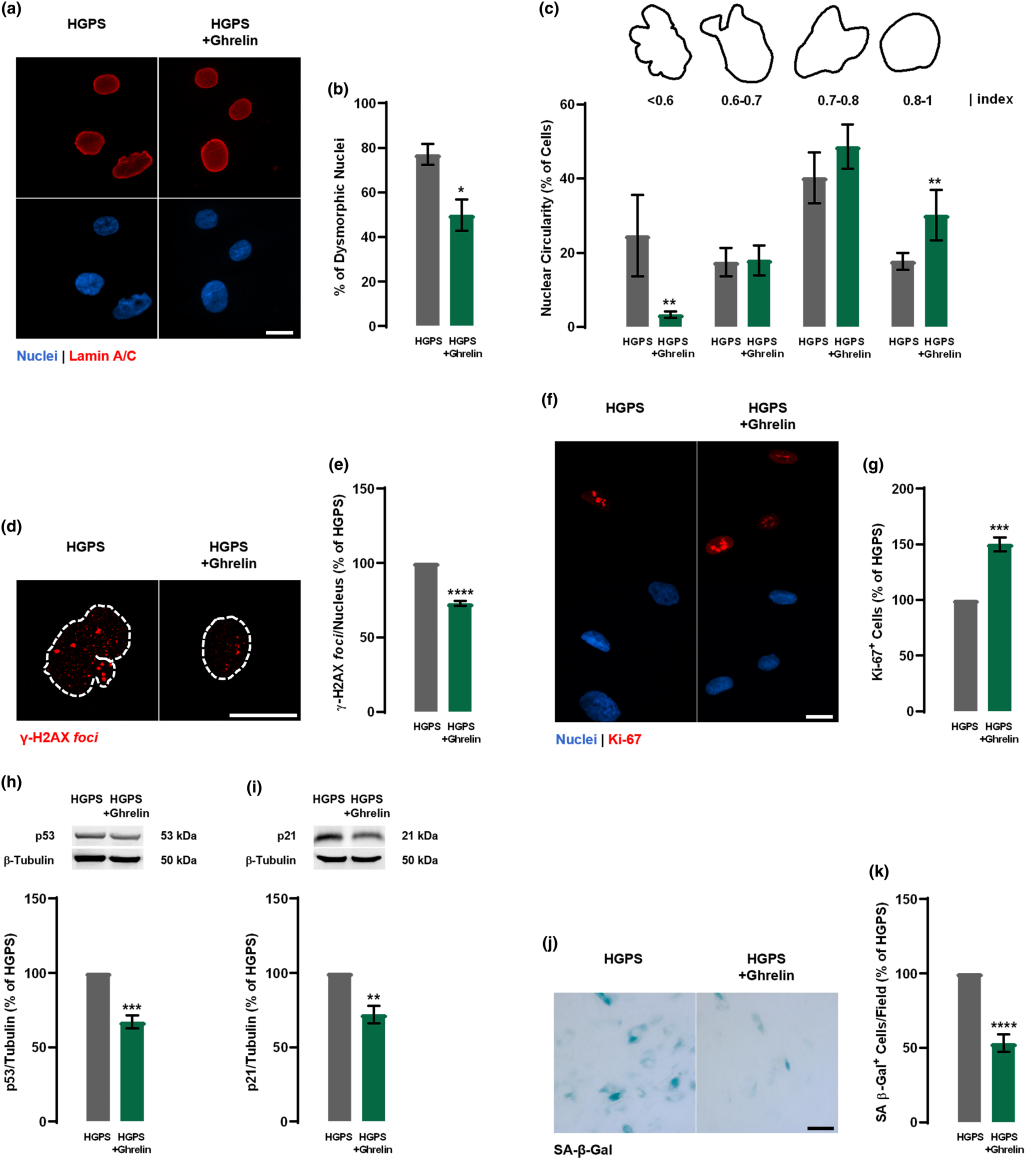
Ghrelin delays cellular senescence in HGPS fibroblasts. (a–k) HGPS fibroblasts were exposed to ghrelin (1 nM; HGPS + Ghrelin) for 1 week for every other day. HGPS‐untreated cells were used as control (HGPS). (a–c) HGPS fibroblasts were immunolabeled for lamin A/C (red, top panel) and nuclei were stained with Hoechst 33342 (blue, bottom panel) (a). Images are representative of four independent experiments. Scale bar, 10 μm. Quantification of the number of misshapen nuclei (b) and nuclear circularity (c) upon ghrelin treatment (*N* = 4–6). For each condition, an equal number of nuclei (>400) were randomly analyzed. Circularity (defined as 4*π*area/perimeter^2^) was measured using ImageJ. A circularity value equal to 1 corresponds to perfectly circular nuclei. (d and e) Ghrelin decreased γ‐H2AX immunoreactivity. Cells were immunolabeled for γ‐H2AX (red). Images are representative of three independent experiments. Scale bar, 20 μm. (e) Quantification of γ‐H2AX *foci* number using ImageJ analysis and customized macros of three independent experiments; >200 cells analyzed). The average number of γ‐H2AX *foci per* nucleus. (f and g) Ghrelin increases cell proliferation, as determined by Ki‐67 immunoreactivity. (f) Cells were immunolabeled for Ki‐67 (red, top panel) and nuclei were stained with Hoechst (blue, bottom panel). Images are representative of four independent experiments. Scale bar, 10 μm. (g) Quantification of the number of Ki‐67‐positive cells in HGPS and ghrelin‐treated HGPS cells. (h and i) Whole‐cell extracts were assayed for p53 (H) (*N* = 4), p21 (*I*) (*N* = 3), and β‐tubulin (loading control) immunoreactivity through western blotting analysis. Representative western blots for each protein are presented above each respective graph. (j and k) Ghrelin decreases cellular senescence, as determined by SA‐β‐Gal activity. (j) Images are representative of five independent experiments. Scale bar, 100 μm. (k) Quantification of SA‐β‐Gal‐positive cells. Data are expressed as the mean ± SEM, at least, three independent experiments, and are expressed as a percentage of HGPS. *p < 0.05, **p < 0.01, ***p < 0.001 and ****p < 0.0001, significantly different from HGPS, as determined by Student's *t* test. HGPS, Hutchinson‐Gilford progeria syndrome.

Given that ghrelin promotes progerin clearance, we further investigated ghrelin potential to rescue other cellular aging hallmarks of HGPS. After 1 week, ghrelin‐treated HGPS cells exhibited a lower number of dysmorphic nuclei (Figure 2a,b), one of several cellular defects in HGPS cells (Cao et al., 2007; Goldman et al., 2004). Furthermore, ghrelin decreased the frequency of nuclei with aberrant circularity (circularity < 0.6; 24.62 ± 10.92% in HGPS vs. 3.29 ± 0.82% in HGPS + Ghrelin) and an increased in the frequency of normal‐shaped nuclei (circularity > 0.8; 17.73 ± 2.32% in HGPS vs. 30.17 ± 6.81% in HGPS + ghrelin; Figure 2c), suggesting that ghrelin, likely through the prevention of progerin accumulation, improves abnormal nuclear morphology of HGPS cells. Noteworthy, ghrelin also improved nuclear circularity and decreased the number of dysmorphic nuclei in primary cultures of fibroblasts from a healthy individual (Figure S5a–c). We next evaluated the effect of 1 week ghrelin treatment on DNA damage through evaluation of the γ‐H2AX *foci*, a DNA damage marker. As shown in Figure 2d, ghrelin treatment decreased γ‐H2AX immunoreactivity and the number of γ‐H2AX *foci* in HGPS cells (Figure 2e). These observations suggest that ghrelin decreases DNA damage in HGPS cells, probably by decreasing progerin accumulation.

### 2.3 Ghrelin stimulates cell proliferation and delays cellular senescence in HGPS cells

Through assessment of Ki‐67 immunoreactivity, we observed that 50% of 1‐week‐ghrelin‐treated HGPS cells showed positivity for this cell proliferation marker, compared to non‐treated HGPS cells (Figure 2f,g). This cell proliferation rescue was also observed in ghrelin‐treated fibroblasts from a healthy individual (Figure S5d,e). The ghrelin‐induced proliferative capacity of HGPS cells was accompanied by downregulation of p53 (Figure 2h) and its downstream effector p21 (Figure 2i), well‐known cell cycle repressors (Varela et al., 2005). Moreover, 1‐week ghrelin‐treated HGPS cells also showed 50% lower senescence‐associated‐β‐Galactosidase (SA‐β‐Gal) activity (Figure 2j,k). These results indicate that ghrelin may increase cell proliferation and postpone cellular senescence in HGPS cells by inhibiting the activation of the p53/p21 signaling pathway.

Overall, the results presented herein provide the basis for a model where ghrelin, via enhancement of autophagy, promotes progerin degradation and ameliorates several cellular defects typically associated with HGPS, including aberrant nuclear architecture, DNA damage, and cellular senescence.

### 2.4 Ghrelin administration ameliorates age‐dependent weight loss and extends the lifespan of *Lmna* ^G609G^ ^/G609G^ mice

**FIGURE 3:**
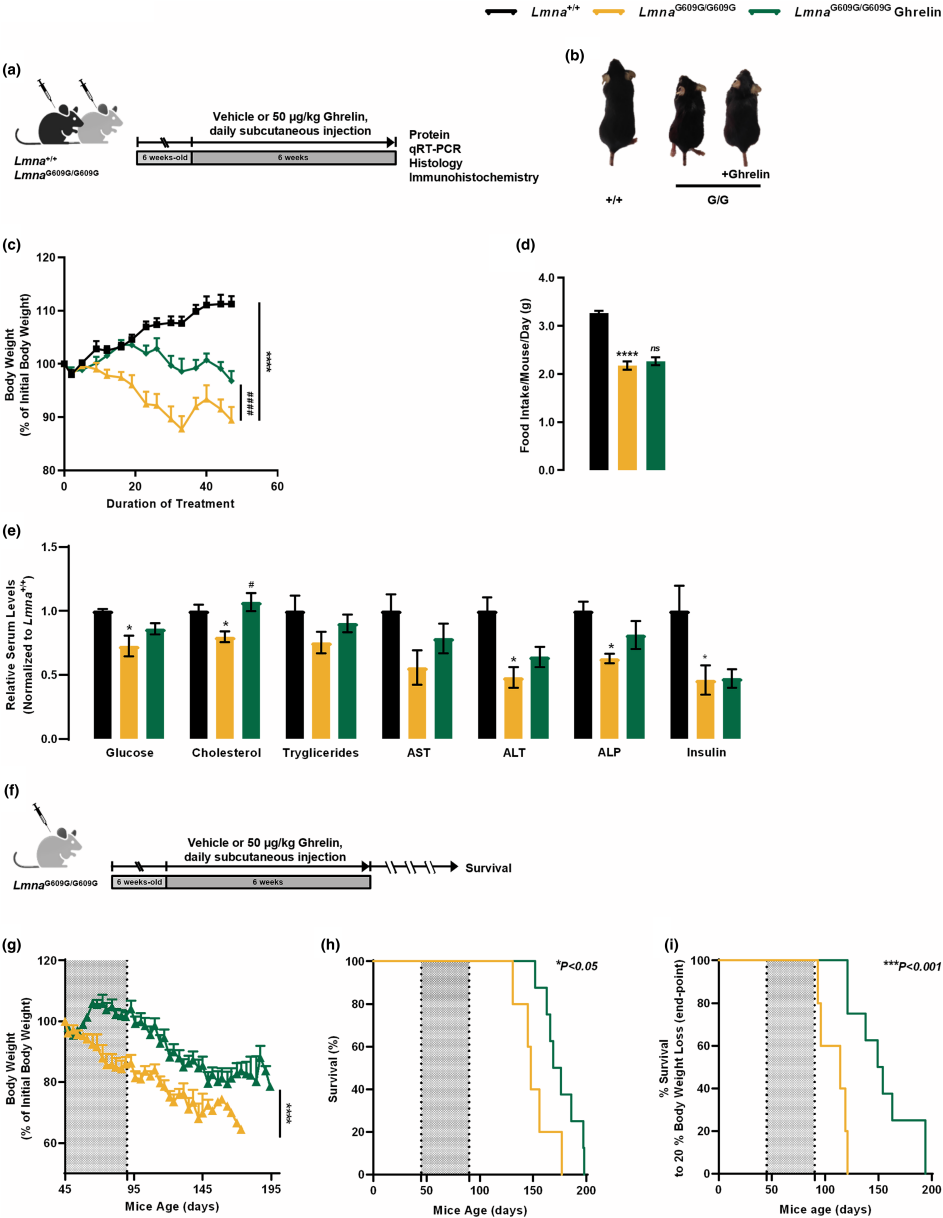
Ghrelin treatment directly affects *Lmna* ^G609G/G609G^ phenotype improving the overall metabolic status without food intake changes, extending lifespan, and preventing age‐associated phenotypes. (a) Schematic representation of the animal protocol. Effect of daily peripheral administration of ghrelin in *Lmna* ^G609G/G609G^ mice premature aging phenotype. (b) Representative photographs of 3 months‐old vehicle‐ or ghrelin‐treated *Lmna* ^+/+^ and *Lmna* ^G609G/G609G^ mice. (c) Cumulative body weight gain of vehicle‐ and ghrelin‐treated *Lmna* ^+/+^ and *Lmna* ^G609G/G609G^ mice, as the percentage of weight gain between the beginning and the end of the study. (d) Daily food intake (in grams) of vehicle‐ and ghrelin‐treated *Lmna* ^+/+^ and *Lmna* ^G609G/G609G^ mice, expressed as g/day. (e) Serum concentration levels of glucose, cholesterol, triglycerides, aspartate transaminase (AST), alanine transaminase (ALT), alkaline phosphatase (ALP) and insulin of vehicle‐ and ghrelin‐treated *Lmna* ^+/+^ and *Lmna* ^G609G/G609G^ mice. Serum concentrations were normalized to the mean of *Lmna* ^+/+^. Data are expressed as the mean ± SEM. *N* = 4–19 *per* group. *p < 0.05 and ****p < 0.0001, significantly different from *Lmna* ^+/+^ mice; ^#^ *p* < 0.05 and ^###^ *p* < 0.001, significantly different compared to *Lmna* ^G609G/G609G^ mice, as determined by one analysis of variance, followed Tukey's multiple comparison test. (f) Schematic representation of the animal protocol. Effect of daily peripheral administration of ghrelin in *Lmna* ^G609G/G609G^ mice health decline. (g) Cumulative body weight gain of vehicle‐ and ghrelin‐treated *Lmna* ^*G609G/G609G*^ mice, as the percentage of weight gain between the beginning and the end of the study. (h) Kaplan–Meier survival plots for vehicle‐ and ghrelin‐treated *Lmna* ^G609G/G609G^ mice. (i) Ghrelin‐treated *Lmna* ^G609G/G609G^ mice show a 37% increased median age at end‐point, compared to vehicle‐treated *Lmna* ^G609G/G609G^ (152 days vs. 114 days respective median age at end‐point; based on mice being terminated upon reaching a 20% body weight loss); and more than 73 days between the longest lived (20% body weight loss) ghrelin‐treated *Lmna* ^G609G/G609G^ and the longest lived vehicle‐treated *Lmna* ^G609G/G609G^ mouse. Data are expressed as the mean ± SEM. *N* = 5–8 per group. *p < 0.05 and ***p < 0.001, significantly different compared to *Lmna* ^G609G/G609G^ mice, as determined by Student's *t* test and Log‐rank/Mantel‐Cox test; chi‐square 5.19 and 11.11 for graphs (h) and (i), respectively.

*Lmna* ^G609G/G609G^ mice, generated by Osorio et al. (2011), carry the c.1827C > T;p.Gly609Gly mutation equivalent to the mutation found in HGPS patients. These mice recapitulate several features of HGPS, including progerin accumulation, reduced growth rate, low body weight, lipodystrophy, bone, and cardiovascular abnormalities, dysregulation of glucose and lipid metabolism, and shortened lifespan (Osorio et al., 2011). Considering the beneficial effects of ghrelin observed in our in vitro model of HGPS (fibroblasts from HGPS patients), we next investigated if ghrelin could rescue the age‐dependent phenotype in *Lmna* ^G609G/G609G^ mice. For this purpose, we administered ghrelin daily to *Lmna* ^G609G/G609G^ and *Lmna* ^+/+^ littermates at 6 weeks of age, for 6 weeks (Figure 3a). Recapitulating previous reports, *Lmna* ^G609G/G609G^ mice exhibited a healthy appearance for 3 weeks after birth, after which they started to show a reduction in growth rate with progressive body weight loss (Figure 3b,c). Ghrelin treatment increased body weight and attenuated body weight loss at later stages of *Lmna* ^*G609G/G609G*^ mice, in contrast to vehicle‐treated mice (Figure 3c). The ghrelin‐induced impact on body weight was independent of food intake (Figure 3d). Additionally, ghrelin‐treated *Lmna* ^G609G/G609G^ mice showed an overall healthier appearance (Figure 3b). Progeroid *Lmna* ^G609G/G609G^ mice exhibited altered circulating plasma concentrations of several hormones and other biochemical markers, as well as low blood glucose levels (Figure 3e). Ghrelin treatment induced a modest, but generalized, improvement in *Lmna* ^G609G/G609G^ mice blood profile, especially in blood glucose and cholesterol levels (Figure 3e). The organ size in progeroid mice was proportional to their reduced body weight (Figure S6a); however, with a significant decrease in liver and spleen weight (Figure S6a). Furthermore, these peripheral organs exhibited several histopathological alterations. Hepatocytes of progeroid *Lmna* ^G609G/G609G^ mice liver were smaller and irregular, with reduced lipid vacuoles, compared to *Lmna* ^+/+^ mice (Figure S6b, upper panel). Moreover, the lumen of sinusoidal capillaries is larger in progeroid *Lmna* ^G609G/G609G^ mice than in *Lmna* ^+/+^ (Figure S6b, upper panel). Ghrelin treatment did not affect the liver weight or histological structure of *Lmna* ^G609G/G609G^ mice (Figure S6a). Masson's trichrome staining was performed in liver sections to assess if there were alterations in collagen levels induced by the accelerated aging process and to evaluate the effect of ghrelin on collagen deposition. We observed lower collagen staining in *Lmna* ^G609G/G609G^ mouse liver, more prominent around the blood vessels, compared to *Lmna* ^+/+^ mice (Figure S6b, bottom panel). Ghrelin treatment reestablished collagen levels in *Lmna* ^G609G/G609G^ mice. Others have described a marked splenic involution in progeroid *Lmna* ^G609G/G609G^ mice compared to wild‐type controls, a feature that was associated with defective immune system of these animals (Osorio et al., 2011). Similarly, we observed lighter spleens in *Lmna* ^G609G/G609G^ mice than wild‐type littermates. Ghrelin‐treated *Lmna* ^G609G/G609G^ mice showed a higher spleen weight compared to *Lmna* ^+/+^ mice (Figure S6a). The histological structure of spleen was not significantly different between both genotypes, except for the white pulp area (Figure S6c,d) which was smaller in *Lmna* ^G609G/G609G^ mice and rescued upon ghrelin treatment (Figure 2c and Figure S6d). The reduced body weight of progeroid *Lmna* ^G609G/G609G^ mice can be aggravated or associated with skeletal muscle atrophy. To investigate possible alterations in the skeletal muscle of *Lmna* ^G609G/G609G^ mice and ghrelin treatment on this tissue, we measured the cross‐sectional area of the muscular fibers. We did not observe significant differences in skeletal muscle histological structure in *Lmna* ^G609G/G609G^ mice compared to *Lmna* ^+/+^ mice, which showed polygonal or round muscle fibers with peripheral nuclei (Figure S6e). However, the cross‐sectional area of skeletal muscle fibers was smaller in *Lmna* ^G609G/G609G^ mice, when compared to *Lmna* ^+/+^ mice (Figure S6e,f). Interestingly, ghrelin treatment rescued the cross‐sectional area of skeletal muscle fibers in *Lmna* ^G609G/G609G^ to the levels observed in *Lmna* ^+/+^ mice (Figure S6e,f). These data demonstrate that ghrelin treatment ameliorates age‐related alterations in peripheral organs, such as the liver, spleen, and skeletal muscle.

As previously described, *Lmna* ^G609G/G609G^ mice have a dramatically shortened life expectancy compared to *Lmna* ^+/+^ mice (Osorio et al., 2011). Given the improved weight loss and growth retardation observed in progeroid mice treated with ghrelin, we hypothesized that ghrelin might have a beneficial effect on the lifespan of *Lmna* ^G609G/G609G^ mice. Ghrelin‐treated *Lmna* ^G609G/G609G^ mice showed significant body weight improvement during their lifespan and lower weight loss at later stages of the disease (Figure 3g) exhibiting significantly healthier aspects than vehicle‐treated mice. Moreover, ghrelin treatment extended the lifespan of progeroid *Lmna* ^G609G/G609G^ mice (Figure 3h,i). The mean survival time of ghrelin‐treated *Lmna* ^G609G/G609G^ mice increased from 148 to 173 days and the maximum survival time increased from 177 to 198 days, representing ~22% increase in the Kaplan–Meier area under the curve (AUC; Figure 3h). The increase in progeroid mice lifespan may relate with delayed body weight loss upon ghrelin treatment. In fact, when the Kaplan–Meier AUC is adjusted for 20% body weight loss, the beneficial effects of ghrelin were even more noticeable between ghrelin‐treated *Lmna* ^G609G/G609G^ mice versus the vehicle‐treated *Lmna* ^G609G/G609G^ mice (Figure 3i). Indeed, we observed a 73‐day gap between the longest lived *Lmna* ^G609G/G609G^ ghrelin‐treated mouse, based on 20% body weight loss, and the longest lived *Lmna* ^G609G/G609G^ vehicle‐treated mouse (Figure 3i). Overall, our results show that ghrelin treatment promotes considerable benefits to the healthspan of progeroid mice, as shown by the effects on age‐dependent body weight loss in *Lmna* ^G609G/G609G^ mice, extending lifespan.

### 2.5 Ghrelin ameliorates aging markers in the aorta and heart of *Lmna* ^G609G^ ^/G609G^ mice

**FIGURE 4:**
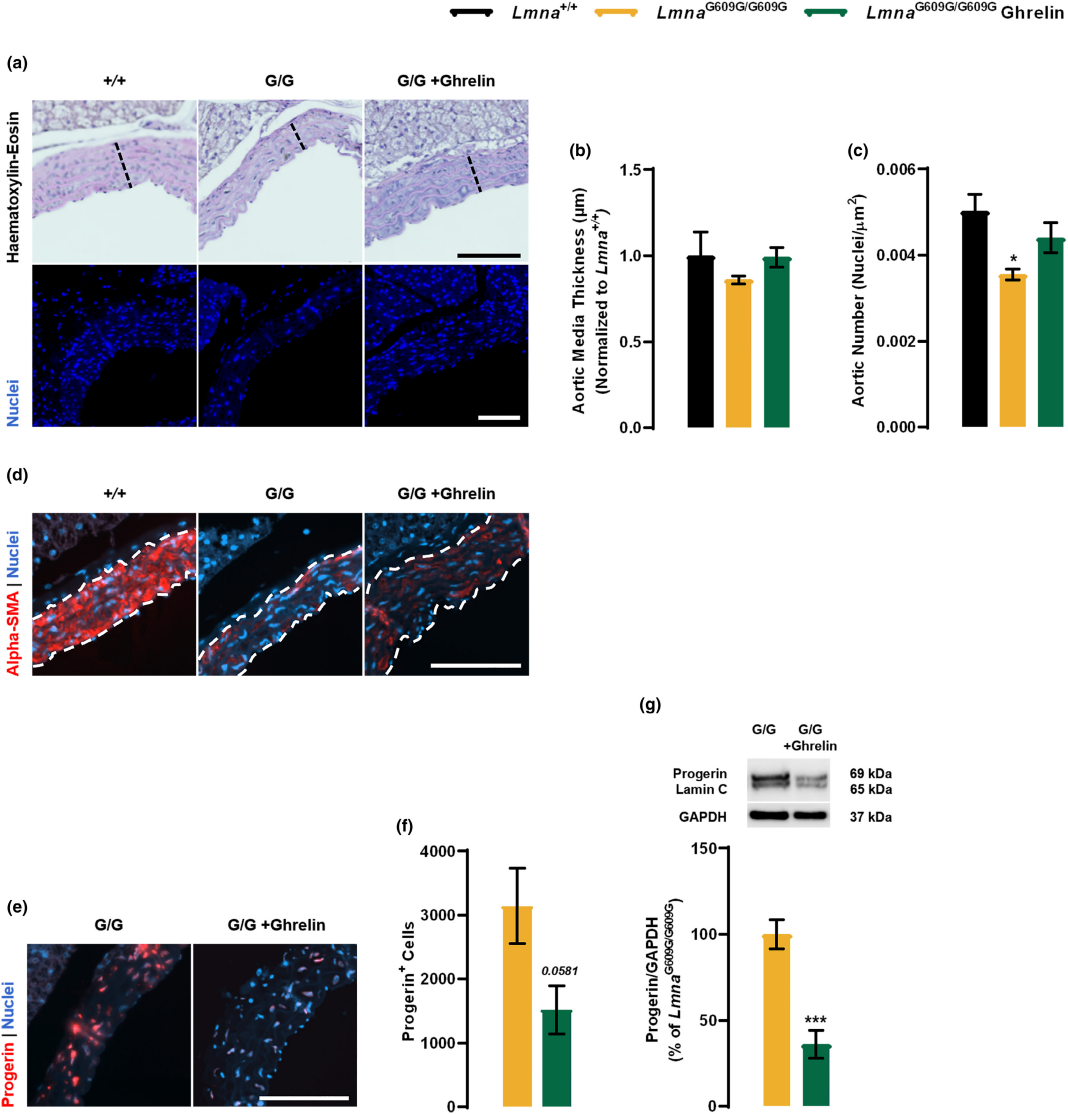
Ghrelin ameliorates cardiac‐related pathology of *Lmna* ^G609G/G609G^ mice. (a) Representative images of Hematoxylin–eosin‐ (top panel) and Hoechst 33342‐ (blue, bottom panel) stained cross‐sections of the aorta of vehicle‐ and ghrelin‐treated *Lmna* ^+/+^ and *Lmna* ^G609G/G609G^ mice. Scale bar, 100 μm. (b and c) Quantification of aortic media wall thickness, expressed in μm (b), and aortic wall nuclei number, expressed as aortic wall nuclei number/mm^2^ (c) in the aorta of vehicle‐ and ghrelin‐treated *Lmna* ^+/+^ and *Lmna* ^G609G/G609G^ mice. (d) Representative images of aorta sections of vehicle‐ and ghrelin‐treated *Lmna* ^+/+^ and *Lmna* ^G609G/G609G^ mice immunolabeled for alfa‐smooth muscle Actin, α‐SMA, (red). Nuclei are stained with Hoechst 33342 (blue). Scale bar, 100 μm. (e) Representative images of the aorta of vehicle‐ and ghrelin‐treated *Lmna* ^+/+^ and *Lmna* ^G609G/G609G^ mice immunolabeled for progerin (red). Nuclei are stained with Hoechst 33342 (blue). Scale bar, 100 μm. (f) Quantification of progerin‐positive cells in the aorta expressed in cell/mm^2^. (g) Heart whole protein lysates were assayed for lamin A/progerin/lamin C and GAPDH (loading control) immunoreactivity through western blotting analysis. The results are expressed as a percentage of *Lmna* ^G609G/G609G^ mice. Representative western blots for each protein are presented in the graph. Data are expressed as the mean ± SEM. *N* = 4–6 *per* group. *p < 0.05, significantly different from *Lmna* ^+/+^ mice, ***p < 0.001, significantly different compared to *Lmna* ^G609G/G609G^ mice, as determined by analysis of variance, followed Tukey's multiple comparison test or Student's *t* test.

The cardiovascular system is severely affected in HGPS patients, with myocardial infarction being the most common cause of death (Ullrich & Gordon, 2015). *Lmna* ^G609G/G609G^ mice recapitulate, to some degree, the abnormalities described in these patients, as they exhibit a depletion of vascular smooth muscle cells (VSMCs) in the aortic arch, which may underlie the cardiovascular system dysfunction (Osorio et al., 2011). We observed a slight but nonsignificant decrease in medial aortic thickness (Figure 4a (upper panel), b) and a decreased cell number in this structure of *Lmna* ^G609G/G609G^ mice (Figure 4a (bottom panel), c), compared with *Lmna* ^+/+^ mice. Ghrelin treatment promoted a slight increase in both aortic thickness (Figure 4a (upper panel), b) and cell density (Figure 4a (bottom panel), c). Progerin induces a dramatic effect on VSMCs, leading to decreased cell viability and increased arterial stiffness (Del Campo et al., 2019). We observed a decrease in alpha‐smooth muscle actin (α‐SMA) immunoreactivity in the aortic wall of *Lmna* ^G609G/G609G^ mice compared to *Lmna* ^+/+^ mice (Figure 4d), which correlates with the loss of cellularity (Figure 4a,c). Ghrelin treatment slightly increased α‐SMA immunoreactivity in *Lmna* ^G609G/G609G^ mice (Figure 4d). We also observed a strong progerin immunoreactivity encircling the nuclei of *Lmna* ^G609G/G609G^ mice (Figure 4e), that was decreased upon ghrelin treatment (Figure 4e,f). Additionally, no histological changes were observed in the heart, between the two genotypes, nor any effect deriving from ghrelin treatment (data not shown). Nevertheless, the hearts of *Lmna* ^G609G/G609G^ mice treated with ghrelin showed lower progerin levels (Figure 4g). These results show that ghrelin prevents VSMC loss and improves aortic structure in *Lmna* ^G609G/G609G^ mice, which may be related to the ghrelin‐induced decrease in progerin levels. Ghrelin might also have beneficial effects on heart functions by lowering progerin levels.

### 2.6 Ghrelin ameliorates skin thinning and subcutaneous adipose tissue atrophy in *Lmna* ^G609G^ ^/G609G^ mice

**FIGURE 5:**
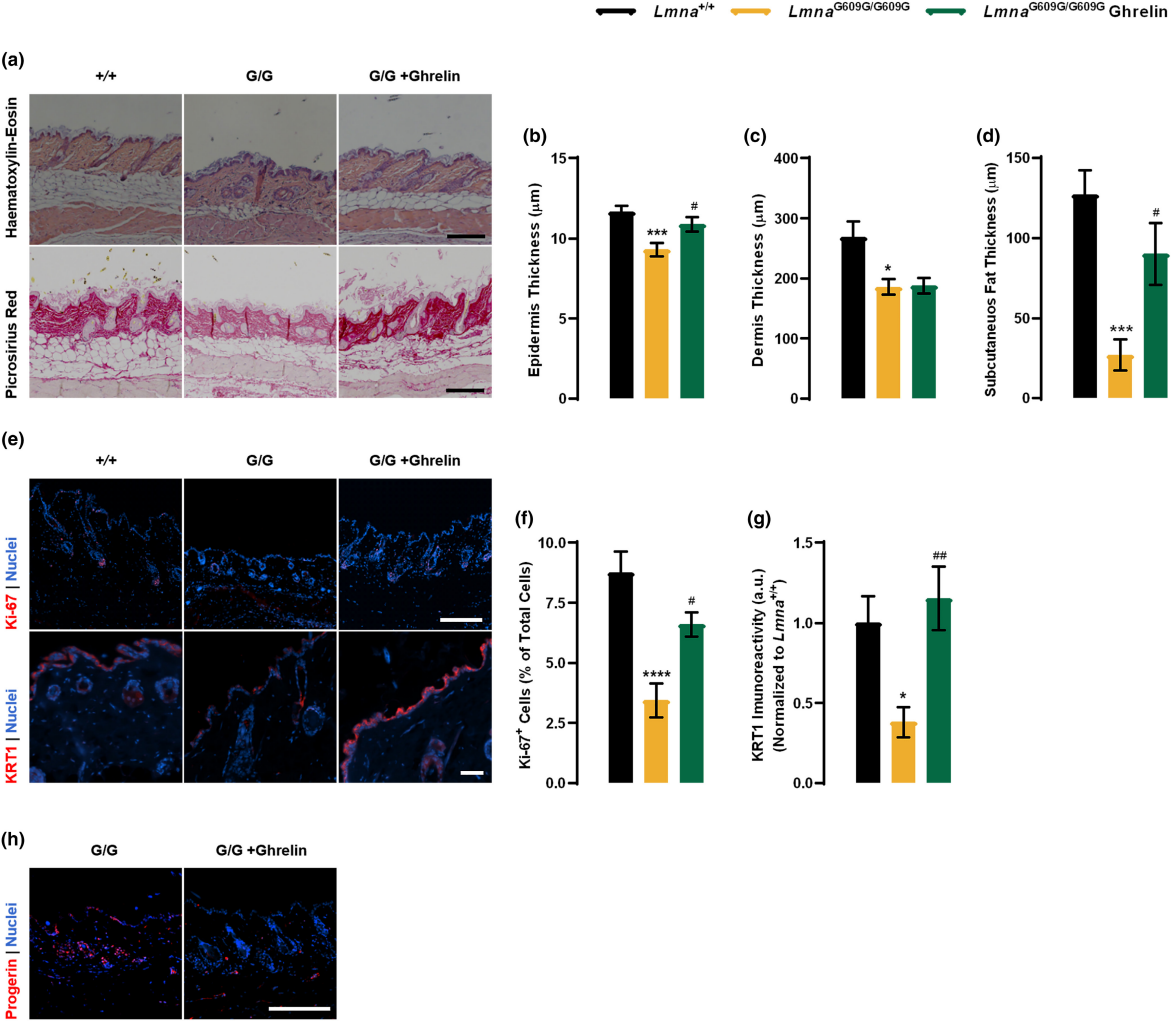
Ghrelin delays skin age‐related alterations in the skin of *Lmna* ^G609G/G609G^ mice. (a) Representative images of Hematoxylin–eosin‐stained (top panel) and Picro‐Sirius‐Red‐stained (bottom panel) sections of dorsal skin of vehicle‐ and ghrelin‐treated *Lmna* ^+/+^ and *Lmna* ^G609G/G609G^ mice. Scale bar, 100 μm. (b–d) Quantification of the epidermis (b), dermis (c) and subcutaneous falt layer thickness (d) (expressed in μm), of the dorsal skin of vehicle‐ and ghrelin‐treated *Lmna* ^+/+^ and *Lmna* ^G609G/G609G^ mice. (e) Representative images of dorsal skin of vehicle‐ and ghrelin‐treated *Lmna* ^+/+^ and *Lmna* ^G609G/G609G^ mice immunolabeled for Ki‐67 (red, top panel) and/or Keratin‐1 (red, bottom panel). Nuclei are stained with Hoechst 33342 (blue). Scale bar, 100 μm. (f) Quantification of Ki‐67‐positive cells in the epidermal layer of the skin, expressed in % of total cells. (g) Quantification of KRT1 immunoreactivity in the epidermal layer of the skin, expressed in a.u, normalized to the mean of *Lmna* ^+/+^. (h) Representative images of dorsal skin sections of vehicle‐ and ghrelin‐treated *Lmna* ^+/+^ and *Lmna* ^G609G/G609G^ mice immunolabeled for progerin (red). Nuclei are stained with Hoechst 33342 (blue). Scale bar, 100 μm. Data are expressed as the mean ± SEM. *N* = 8–14 per group. *p < 0.05, ***p < 0.001 and ****p < 0.0001, significantly different from *Lmna* ^+/+^; ^#^ *p* < 0.05 and ^##^ *p* < 0.01, significantly different compared to *Lmna* ^G609G/G609G^ mice, as determined by analysis of variance, followed Tukey's multiple comparison test.

In HGPS patients, skin presents relevant alterations: hyperpigmentation and hypopigmentation, gradual loss of the subcutaneous fat layer, which makes the skin extremely thin, prominent blood vessels, particularly those on the face and scalp, and loss of hair (Hennekam, 2006; Ullrich & Gordon, 2015). Others described some of these skin features in *Lmna* ^G609G/G609G^ mice, namely the decrease in the subcutaneous fat layer and hair follicle attrition (Osorio et al., 2011). Using histomorphometry analysis of dorsal skin (Figure 5a, upper panel) we observed that *Lmna* ^G609G/G609G^ mice exhibited thinning of the epidermis compared to *Lmna* ^+/+^, a feature rescued with ghrelin treatment (Figure 5b). Furthermore, progeroid *Lmna* ^G609G/G609G^ mice showed a thinner dermis when compared to *Lmna* ^+/+^ mice; however, ghrelin treatment had no impact on this structure (Figure 5c). *Lmna* ^G609G/G609G^ mice showed severe thinning and atrophy of the subcutaneous fat layer, the hypodermis, (Figure 5d). Ghrelin‐treated *Lmna* ^G609G/G609G^ mice displayed a thicker subcutaneous adipose layer, suggesting that ghrelin ameliorates the lipodystrophic phenotype of progeroid mice (Figure 5d). The degradation of collagen fibers is one of the major age‐associated alterations in the skin. In comparison to *Lmna* ^+/+^ mice, the skin of *Lmna* ^G609G/G609G^ mice contained lower levels of collagen (Figure 5a, bottom panel), and the collagen bundles appear to be organized in a similar fashion with open spaces between the collagen fibers. However, treatment with ghrelin increased collagen deposition in *Lmna* ^G609G/G609G^ mice, suggesting that ghrelin may promote collagen synthesis or remodeling (Figure 5a). To evaluate the proliferative capacity of skin cells, we used Ki‐67 immunostaining and observed a lower number of Ki‐67‐positive cells in the skin of the progeroid mice (Figure 5e, upper panel); however, the Ki‐67‐positive cells were arranged in niches, especially in the hair follicles bulb and in some regions of the epidermis (Figure 5f). Ghrelin treatment increased the number of Ki‐67‐positive cells in these areas, indicating that ghrelin promotes proliferation in the epidermis of the *Lmna* ^G609G/G609G^ mice skin (Figure 5f). We also evaluated skin structure by examining the immunoreactivity of Keratin‐1 (KRT1), a critical marker of skin integrity (Figure 5f, bottom panel). The skin of *Lmna* ^G609G/G609G^ mice exhibited lower KRT1 immunoreactivity compared to *Lmna* ^+/+^ mice (Figure 5g); however, this was improved with ghrelin treatment, suggesting that ghrelin can improve skin structure and integrity. Additionally, we observed strong progerin immunoreactivity surrounding the nuclei of skin cells in *Lmna* ^G609G/G609G^ mice. Ghrelin treatment decreased progerin immunoreactivity and/or the density of progerin‐positive cells (Figure 5h). Overall, these findings indicate that ghrelin can ameliorate age‐related changes in the skin of *Lmna* ^G609G/G609G^ mice.

### 2.7 Ghrelin rescues the lipodystrophic phenotype of *Lmna* ^G609G^ ^/G609G^ mice by reverting progerin‐affected genes in the adipogenic network

**FIGURE 6:**
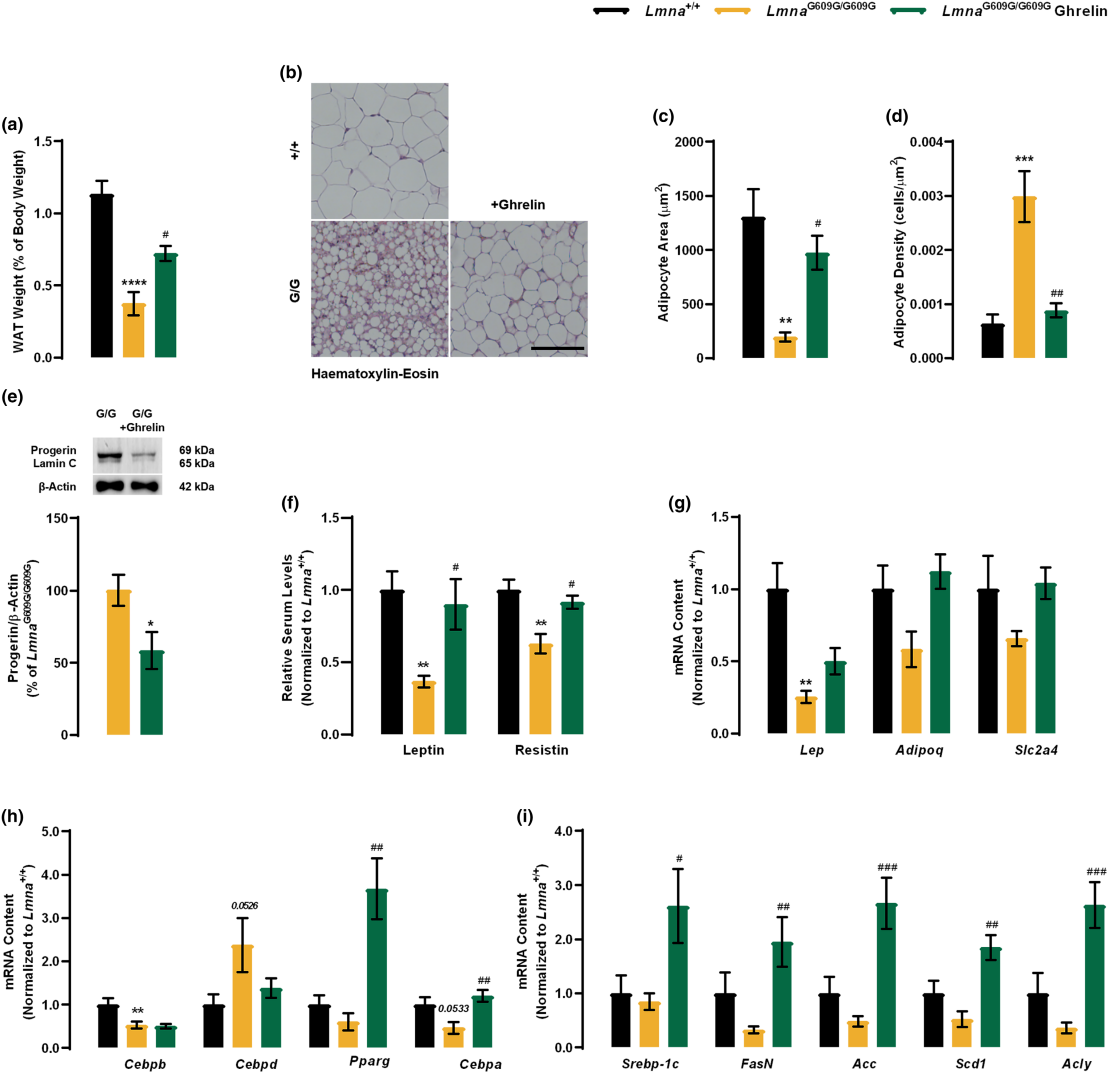
Ghrelin alleviates pathological changes in fat distribution and reverts the progerin‐impacted transcription of core adipogenic regulators during adipocyte differentiation of *Lmna* ^G609G/G609G^. (a) Size of the gonadal white adipose tissue (WAT), expressed as a percentage of % of body weight in 3 months‐old vehicle‐ and ghrelin‐treated *Lmna* ^+/+^ and *Lmna* ^G609G/G609G^ mice. (b) Representative images of hematoxylin–eosin‐stained sections of WAT of vehicle‐ and ghrelin‐treated *Lmna* ^+/+^ and *Lmna* ^G609G/G609G^ mice. Scale bar, 100 μm. (c) Quantification of adipocyte area (μm^2^) of vehicle‐ and ghrelin‐treated *Lmna* ^+/+^, and *Lmna* ^G609G/G609G^ mice. (d) Quantification of adipocyte density (cells/μm^2^) of vehicle‐ and ghrelin‐treated *Lmna* ^+/+^ and *Lmna* ^G609G/G609G^ mice. (e) WAT protein lysates were assayed for lamin A/progerin/lamin C and β‐Actin (loading control) immunoreactivity through western blotting analysis. The results are expressed as a percentage of *Lmna* ^G609G/G609G^ mice. Representative western blots for each protein are presented in the graph. (f) Serum concentration levels of leptin and resistin of vehicle‐ and ghrelin‐treated *Lmna* ^+/+^ and *Lmna* ^G609G/G609G^ mice. Serum concentrations were normalized to the mean of *Lmna* ^+/+^. (g–i) Quantitative polymerase chain reaction analysis mRNA levels of (g) *Lep, Adipoq* and *Slc2a4* (h) WAT adipogenic differentiation genes (*Cebpb*, *Cebpd* (early differentiation regulators), *Pparγ* and *Cebpab* (late differentiation regulators)), (i) lipogenic genes (*Srebp‐1c*, *FasN, Acc, Scd1* and *Acly*), fatty acid β‐oxidation (*Mcad* and *Cpt1*) and gluconeogenic gene (*Pepck*), mRNA contents were normalized to the mean of *Lmna* ^+/+^ mice. Data are expressed as the mean ± SEM. *N* = 5–12 per group. *p < 0.05, **p < 0.01 and ****p < 0.0001, significantly different from *Lmna* ^+/+^ or *Lmna* ^G609G/G609G^ mice; ^#^ *p* < 0.05, ^##^ *p* < 0.01 and ^##^ *p* < 0.01, significantly different compared to *Lmna* ^G609G/G609G^ mice, as determined by analysis of variance, followed Tukey's multiple comparison test or Student's *t* test.

*LMNA* mutations are associated with lipodystrophic features, which combine generalized or partial fat atrophy and metabolic alterations that could result from altered adipocyte differentiation or altered fat structure (Bidault et al., 2011; Boguslavsky et al., 2006), features also observed in *Lmna* ^G609G/G609G^ mice (Osorio et al., 2011). Here, we observed a significantly lower gonadal white adipose tissue (WAT) weight in *Lmna* ^G609G/G609G^ mice compared with *Lmna* ^+/+^ mice, which was reversed upon ghrelin treatment (Figure 6a). Histomorphometry analysis of gonadal fat also revealed significantly reduced adipocyte area in *Lmna* ^G609G/G609G^ mice (Figure 6b,c). Concomitant with a WAT increase, ghrelin treatment induced an increase in adipocyte cross‐sectional area, as well as a decrease in adipocyte density, due to the increased adipocyte size in *Lmna* ^G609G/G609G^ mice (Figure 6c,d), rescuing WAT fibrosis in progeroid mice (Figure 6b). Progerin accumulation results in metabolic dysfunction, one crucial hallmark of the progeria phenotype, and ghrelin treatment decreased progerin protein levels in *Lmna* ^G609G/G609G^ mice (Figure 6e). Regarding brown adipose tissue (BAT), *Lmna* ^G609G/G609G^ mice showed a lower quantity of BAT, when compared to *Lmna* ^+/+^ (Figure S6g). with smaller adipocytes and with fewer lipid inclusions (Figure S6h). Ghrelin treatment rescued not only *Lmna* ^G609G/G609G^ BAT weight (Figure 6g) but also BAT architecture (Figure S6h). As in WAT, BAT of ghrelin‐treated *Lmna* ^G609G/G609G^ mice showed lower progerin levels compared to untreated‐progeroid mice (Figure S6i). The structural abnormalities observed in the WAT of *Lmna* ^G609G/G609G^ mice may be caused by an impairment of the adipogenic process, which may significantly impact the endocrine function of this organ and consequently regulate body weight and metabolism. We next investigated how ghrelin impacts WAT function by analyzing the levels of relevant hormones produced by WAT – leptin, adiponectin, and resistin. Consistent with their lipodystrophic phenotype, *Lmna* ^G609G/G609G^ mice exhibited lower serum or gene expression of these hormones, a feature that was counteracted through ghrelin treatment (Figure 6f,g).

The adipocyte lifecycle is activated by an initial transient increase of CCAAT‐enhancer‐binding proteins (C/EBP) isoforms β and δ, which, in response to adipogenic signals, precede the induction of peroxisome proliferator‐activated receptor‐gamma (PPARγ) and C/EBPα (Rosen & MacDougald, 2006). In the WAT of *Lmna* ^G609G/G609G^ mice, *Cebpb*, *Ppar*γ, and *Cebpa* were downregulated while *Cebpd* was upregulated compared to *Lmna* ^+/+^ mice. Ghrelin treatment had no impact on *Cebpb* expression, but rescued the expression of *Cebpd*, *Ppar*γ, and *Cebpa* (Figure 6h). Moreover, several enzymes involved in de novo lipogenesis (*Srebp‐1c*, *FasN*, *Acc*, *Scd1* and *Acly*), fatty acid oxidation (*Mcad* and *Cpt1*), and gluconeogenesis (*Pepck*), showed decreased expression in *Lmna* ^G609G/G609G^ mice, which was reverted with ghrelin treatment (Figure 6i). These data indicate that mutations in *Lmna* resulted in adipogenic and metabolic defects. Ghrelin treatment promotes WAT differentiation and maturation and improves WAT function in *Lmna* ^G609G/G609G^ mice concomitant with progerin clearance, which could counteract the lipodystrophy observed in this mouse model.

## 3 DISCUSSION

Hutchinson‐Gilford progeria syndrome (HGPS) is a rare and lethal genetic condition that results in premature and accelerated aging. This disorder arises from a spontaneous point mutation in the *LMNA* gene, which gives rise to a defective prelamin A form, known as progerin (De Sandre‐Giovannoli et al., 2003; Eriksson et al., 2003). HGPS represents a paradigm for translational medicine in aging. The identification of novel therapeutic agents for this disease is crucial and provides promising strategies for delaying the aging process. Numerous treatments have been suggested to improve the HGPS condition, such as rapamycin, sulforaphane, and MG132, which are known to enhance proteostasis and progerin clearance, leading to an improvement of aging‐related defects in HGPS cells (Guilbert et al., 2021). Although promising for treating HGPS in vivo, cautious strategies are still needed before translation into clinical trials.

Rescue of autophagy impairment delays the aging progression of aging in HGPS (Guilbert et al., 2021); therefore ghrelin, a hormone that stimulate autophagy and progerin clearance, could benefit HGPS phenotype. Moreover, ghrelin might also benefit other HGPS features through its known effects on body weight, white adipose tissue, metabolism, cardiovascular function, and bone mass regulation (Kreienkamp & Gonzalo, 2020). Here we show, for the first time, that ghrelin enhances progerin clearance and delays cellular senescence in HGPS cells. Importantly, we demonstrated that ghrelin treatment ameliorates the aging phenotype and extends the lifespan of HGPS mice. These findings suggest that ghrelin could be a promising therapeutic candidate for HGPS and other age‐related diseases. First, we found that ghrelin reduces the accumulation of progerin in HGPS cells by enhancing autophagic flux. This result is in line with our previous reports in rodent neurons showing that ghrelin stimulates autophagy (Ferreira‐Marques et al., 2016, 2021). One of the most notable characteristics of HGPS cells is their abnormal nuclear shape, which results from the accumulation of progerin in the nuclear membrane (Cao et al., 2011). Ghrelin improves nuclear architecture, decreasing the number of deformed nuclei, which can result from the progerin clearance from the nuclear envelope. Consequently, the normal lamin A protein could interact with the nuclear envelope, devoid of progerin, and restore the normal nuclear scaffold, ensuring the stability and integrity of the nucleus of these cells. Ghrelin also reduces DNA damage in HGPS cells; through the decrease of progerin levels, ghrelin may restore the connection between the lamins and DNA which may lead to a reduction in DNA damage and ultimately prevent genomic instability (Lopez‐Otin et al., 2023). Ghrelin also improves the proliferative cell capacity, through decreased activation of the p53/p21 pathway. Chronic activation of p53 due to permanent DNA damage is a common denominator between cell senescence and DNA damage in HGPS patients (Benson et al., 2010; Liu et al., 2006). Ghrelin treatment led to reduced progerin levels and nuclear abnormalities, which triggered the activation of checkpoint response to nuclear abnormalities. This inhibited the stress signaling pathways activated by p53 and enhanced the DNA damage response machinery, thereby promoting reactivation of the cell cycle (Benson et al., 2010; Liu et al., 2006).

Second, we have demonstrated that treating HGPS mice with ghrelin ameliorates the main age‐related histopathological alterations of mesenchymal tissues (Ribeiro et al., 2022), such as lipodystrophy, skin thinning, liver fibrosis, muscle atrophy, and aortic thinning, ultimately attenuating the age‐related functional decline, and extending the health and lifespan of progeria mice. Current evidence suggests that metabolic and endocrine alterations contribute to the HGPS phenotype (Kreienkamp & Gonzalo, 2020). Adipose tissue is an endocrine and metabolic organ that plays an important role in energy homeostasis (Wozniak et al., 2009). It also regulates several body functions, such as blood pressure, reproduction, angiogenesis, and immune response (Wozniak et al., 2009). The progeria mouse model used in this study – *Lmna* ^G609G/G609G^ mice – successfully recapitulates the patient adipose tissue phenotype (Zhang et al., 2013), showing smaller adipocytes that may suggest an impaired capacity to store lipids and may represent one of the causes of lipodystrophy in these animals. One of the major findings of our study is that ghrelin treatment can rescue the structural abnormalities found in the white adipose tissue (WAT) adipocytes and, consequently, WAT loss (severe lipodystrophy) in HGPS mice. This beneficial effect of ghrelin may be explained not only by the well‐described ghrelin action on adiposity and energy expenditure inhibition (Tschop et al., 2000), but also from its direct action on WAT‐promoting adipocyte differentiation, maturation, and lipid accumulation. Capanni et al. (2005) reported an in vivo interaction between prelamin A and SREBP1, a transcription factor upstream of PPARγ and essential for adipocyte differentiation. This interaction resulted in the sequestration of SREBP1 from the nuclear interior to the periphery, leading to the downregulation of its target genes. It is possible that progerin, known to sequester several transcriptional factors at the nuclear periphery through a similar mechanism, may also promote similar change (Ghosh et al., 2015). Based on our findings, we hypothesize that the structural changes we observed in the WAT of *Lmna* ^G609G/G609G^ mice may be caused by an impairment of the adipogenic process. Adipogenesis is controlled by a cascade of fat cell‐related transcriptional factors (Rosen & MacDougald, 2006). We found that C/EBPβ, an early adipogenic gene, as well as PPARγ and C/EBPα, essential for adipogenic differentiation and maturation, were downregulated in *Lmna* ^G6009G/G609G^ mice. Furthermore, ghrelin treatment rescued adipocyte maturation, related to decreased progerin levels and improved adipocyte architecture and function. Ghrelin treatment also restored leptin levels, an important hormone for WAT function (Considine et al., 1996), and the expression of several key regulators of lipogenesis, fatty acid oxidation, and glucose homeostasis in WAT from *Lmna* ^G609G/G609G^ mice. Ghrelin treatment also improved the serum levels of cholesterol, triglycerides, and glucose, improving the overall metabolic status of the mice. However, the increased glucose levels observed may be related to the inhibition of insulin secretion, which has been described because of ghrelin (Dezaki et al., 2004). The effect of ghrelin on blood glucose levels could be a protective mechanism, as it may minimize hypoglycemia by decreasing glucose uptake by different tissues during fasting periods when glucose levels are low. Since progeroid mice show hypoglycemia (Osorio et al., 2011), which could aggravate the cardiovascular abnormalities observed in these animals, the effect of ghrelin on blood glucose levels could have a beneficial impact on cardiovascular functions and minimize the progression of the disease. The skin of HGPS patients is also severely affected by disease progression, resulting in thinner skin and contributing to the lipodystrophic phenotype (Hennekam, 2006). Skin alterations have also been reported in *Lmna* ^G609G/G609G^ mice (Osorio et al., 2011), including loss of the subcutaneous fat layer and wear of hair follicles. We also found tha*t Lmna* ^G609G/G609G^ mice showed an overall thinning of the epidermis, dermis, and hypodermis due to decreased cell proliferative capacity. However, the subcutaneous fat layer changes are more pronounced, probably related to severe lipodystrophy in these animals (Osorio et al., 2011). With the normal aging process, the skin also becomes thinner as the number of epidermal cells decreases, the vascularity and cellularity of the dermis decrease, and the subcutaneous fat in the hypodermis falls (Farage et al., 2013). These processes are exacerbated in HGPS mice, accelerating the aging phenotype. We found that ghrelin treatment restored epidermal layer thinning in *Lmna* ^G609G/G609G^ mice, which may be triggered by increased cell proliferation. One hypothesis to explain the observed beneficial effects of ghrelin treatment is that it restored epidermal layer thinning in *Lmna* ^G609G/G609G^ mice, which may result from increased cell proliferation. Ghrelin may also induce the proliferation of pre‐existing keratinocytes, considering its well‐established role as a cell proliferation stimulator (Yin & Zhang, 2016). Another possible mechanism underlying this effect is the increased stem cell proliferation and differentiation, which has already been described in diverse models (Gao et al., 2013; Moon et al., 2009). Age‐associated skin changes also include the decrease in the extracellular matrix components, such as collagen (Farage et al., 2013). Ghrelin treatment attenuated collagen loss in progeroid mice, suggesting increased collagen synthesis and turnover. However, further studies are required to explore collagen and extracellular matrix alterations in the skin of these mice, given that they are also related to cell proliferative capacity (Hernandez et al., 2010).

Overall, we conclude that, ghrelin improved the healthspan and extended the lifespan of *Lmna* ^G609G/G609G^ mice by improving WAT function, metabolic profile, and increasing body weight in ghrelin‐treated HGPS mice. Our findings provide compelling in vitro and in vivo evidence that ghrelin ameliorates the characteristic changes caused by progerin‐induced premature aging. Ghrelin ameliorates healthspan and extends the lifespan of these mice by improving white adipose tissue structure and function, rescuing the lipodystrophy phenotype. As ghrelin and the fact that ghrelin and its analogues have already been used in several clinical trials as a therapeutic strategy for the treatment of conditions such as cachexia in chronic heart failure, frailty in the elderly, anorexia nervosa, growth hormone deficient patients (Akamizu & Kangawa, 2012; Strasser, 2012), the findings of this study provide ghrelin as an effective intervention to delay premature aging in HGPS and normal cellular aging, thus enhancing healthspan and lifespan.

## 4 MATERIALS AND METHODS

### 4.1 Cell cultures

Primary human dermal fibroblast cell cultures derived from two HGPS patients (HGADFN003, a 3‐year‐old male; and HGADFN127, a 2‐year‐old female) from the Progeria Research Foundation and were used as an in vitro model of HGPS. Primary cultures of human dermal fibroblasts obtained from a skin biopsy of a healthy individual (Control fibroblasts) were also used and provided by Coriell Cell Repositories. The cells were cultured in Dulbecco's modified Eagle's medium (DMEM, 4.5 g/L D‐glucose; Sigma) supplemented with 15% fetal bovine serum (FBS; Gibco), 2 mM L‐glutamine (Gibco) and 100 U/mL penicillin and 100 μg/mL streptomycin (Gibco) and maintained at 37°C and 5% CO_2_/air. All results shown in this article correspond to HGPS fibroblasts HGADFN003. The results related to HGPS fibroblasts HGADFN127 can be found in Figures S3 and S4. We used in previously established non‐HGPS control dermal fibroblast cultures in parallel. Control fibroblasts were used between Passages 14 and 19, whereas HGPS fibroblasts were between 13 and 23.

### 4.2 Experimental conditions

HGPS and Control fibroblasts were treated with human Ghrelin (1 nM; Bachem) every other day for up to 1 week, unless otherwise indicated. The lysosomal protein degradation inhibitor chloroquine (ChQ; 100 μM; Sigma‐Aldrich) was added to the cell culture medium 30 min prior to ghrelin treatment for 6 h.

### 4.3 Western blotting

Western blotting was performed as described in Materials and Methods in Data S1.

### 4.4 Autophagic flux measurement by LC3B turnover assay

The LC3B turnover assay measures the amount of LC3B‐II delivered to the lysosomes by comparing the LC3B‐II amount in the presence and absence of the lysosomal inhibitor chloroquine (ChQ; 100 μM) by Western blotting. For each experimental condition, untreated HGPS cells (used as control) and ghrelin‐treated HGPS cells, “Autophagic flux,” was determined by subtracting the densitometric value of the LC3B‐II band of the chloroquine‐untreated sample (ChQ‐LC3B‐II) from the densitometric value of the LC3B‐II band of the corresponding chloroquine‐treated sample (ChQ + LC3B‐II; Klionsky et al., 2021; Mizushima et al., 2010; Zhang et al., 2016). If autophagic flux is stimulated, the amount of LC3B‐II will be higher in the presence of lysosomal inhibitor (ChQ). Conversely, if the LC3B‐II protein does not increase in the presence of ChQ, it indicates that autophagic flux is not stimulated, and a defect or delay earlier in the process, prior to degradation at the autolysosome, has occurred. For each independent experiment, the values obtained upon subtraction for each condition were normalized to the control condition (untreated HGPS cells). A similar analysis was performed for SQSTM1, also known as p62, an autophagic substrate. The results are represented as mean values for each experimental condition.

### 4.5 Immunocytochemistry

Immunocytochemistry was performed as described in Materials and Methods in Data S1.

### 4.6 Senescence‐associated‐β‐galactosidase (SA‐β‐Gal) assay

Senescence‐associated β‐galactosidase (SA‐β‐Gal) activity in HGPS cells was measured as described previously (Itahana et al., 2013). To quantify SA β‐Gal‐positive cells, 30 randomly chosen non‐overlapping fields were examined for each coverslip of each experimental condition at ×200 magnification. The number of positive cells was directly counted.

### 4.7 Mouse strains and experiments

The mouse model of HGPS carrying the *LMNA* causative mutation p.Gly609Gly (*Lmna* ^G609G/G609G^, with the genetic background of C57BL/6) were used to perform in vivo experimental studies. The infertile *Lmna* ^G609G/G609G^ mice were obtained by crossing heterozygous (*Lmna* ^G609G/+^) females and males. Both male and female animals were used in all experiments. Animals were housed in pairs or fours *per* cage, under a 12‐h:12‐h light/dark cycle, with controlled temperature and humidity and ad libitum access to normal standard chow diet. As the *Lmna* ^G609G/G609G^ mice advanced beyond 3 months of age and in consideration of the logistical challenges related to food and water accessibility, we implemented additional specific procedures. Water in the form of hydrogel was provided, and moistened pellets were placed on the floor daily. This approach aimed to mitigate potential barriers to food and water intake, thereby ensuring the well‐being of the mice and facilitating uninterrupted progress in the study. All experimental work was approved by CNC Animal Welfare Body (ORBEA 329 and DGAV 009428) and performed following the European Community directive for the care and use of laboratory animals (86/609/EEC) and the Portuguese law for the care and use of experimental animals (Decree‐law 113‐2013). The animals were housed in the licensed animal facility of CNC International Animal Welfare Assurance number 520.000.000.2006. Animal experimentation was performed by credited and trained investigators, as required by the Portuguese authorities. The study was included in projects approved and financed by the Progeria Research Foundation, which approved the use of animals for this study (PRF2014‐53 and PRF2015‐60). Ghrelin (Bachem) was administered at a concentration of 50 μg/kg each in 0.9% NaCl through daily subcutaneous injection for 6 weeks. Control mice were administered the same routine as a vehicle, 0.9% NaCl. Survival curve analysis, performed twice (*N* = 9–13), was terminated when animals reached predefined humane endpoints according to the FMUC/CNC‐UC animal facility.

#### 4.7.1 Food intake and body weight analysis

All experiments measured body weight and food intake twice a week. Body weight gain was calculated as a percentage of weight gain and presented in a plot graph as body weight (% of initial body weight). Food intake was presented as the total ingestion of calories within the experimental period. As mice were not kept in individual cages due to rules respecting our animal facility, the individual food intake was calculated as follows: (Total food intake per cage/Total weight per cage) × Mouse weight (g) (Carmo‐Silva et al., 2023).

#### 4.7.2 Tissue and blood collection

Animals were euthanized 6 weeks after starting treatment by sodium pentobarbital overdose. Animals from each experimental group were randomly distributed for blood collection, and peripheral organs and tissues were collected for RNA and/or protein extraction and/or histological analysis. Blood was collected upon decapitation, and serum was obtained by centrifugation (2000 × *g* for 15 min). After decapitation, several organs, and tissues, such as gonadal white adipose tissue (WAT) and interscapular brown adipose tissue (BAT), skin, heart, aorta, liver, skeletal muscle, and spleen, were collected and weighed. These organs were then cut and divided. A part of each organ was kept at −80°C for protein and RNA extraction purposes, while the other was kept in a 10% neutral buffered formalin solution for 48 h to prepare them for histological processing. Serum samples were kept at −20°C until use.

#### 4.7.3 Biochemical parameters assessment

Biochemical profiles were measured on Cobas 6000 from Roche. Glucose levels were measured using a FreeStyle Precision Neo glucometer (Abbott).

#### 4.7.4 Plasma metabolism multiplex assay array

Metabolic hormone levels were measured in mouse serum samples replicates using a MILLIPLEX® Mouse Metabolic Hormone Magnetic Bead Panel, Metabolism Multiplex Assay Array (#MMHMAG‐44K; Millipore). The levels were determined through a multiplex array using the Bio‐PlexTM system (a service provided at 476 Biocant Park).

### 4.8 Gene expression analysis

Total RNA extraction, reverse transcription, and quantitative real‐time PCR (qRT‐PCR) analysis were performed as described in Materials and Methods in Data S1.

### 4.9 Histological analysis

Tissue samples were collected and fixed in 10% formalin, cut into small fragments, and underwent several steps for paraffin embedding: ethanol 70% for 1 h; two series of ethanol 95%, 40 min each; two series of ethanol 100%, 1 h each; two series of xylene, 1 h each and two series of paraffin at 56°C, 1 h each. At the end of this process, tissue samples were included in paraffin blocks. Paraffin blocks were sectioned using a microtome (HM325, Thermo Fisher Scientific), and 3–5 μm thickness sections were placed into microscopy slides until use. Haematoxylin‐eosin staining was performed according to standard procedures. After staining, the sections were mounted on slides with Richard‐Allan Scientific Mounting Medium (Thermo Fisher Scientific). The nuclei were stained blue, and the cytoplasm red to detect structural alterations in the tissue. Masson's trichrome (Thermo Fisher Scientific) and Picro‐Sirius Red (Direct Red 80 dye/Sirius Red F3BA; Sigma Aldrich) staining were performed according to the manufacturer's protocol. Tissue section images were acquired using a Zeiss Axio Imager Z2 microscope (Zeiss) with the Plan‐Apochromat 20×/0.8 M27 objective. The images were analyzed with FIJI (Fiji is Just ImageJ) Software. The analysis was performed by a researcher who was unaware of the experimental groups.

### 4.10 Immunohistochemistry

Protein levels of progerin, alpha‐smooth muscle actin (α‐SMA), Ki‐67, and keratin 1 (KRT1) were accessed by fluorescent immunohistochemistry of formalin‐fixed, paraffin‐embedded skin and/or aorta sections collected on Superfrost slides. Similarly, to the histological staining protocol described in the main manuscript, an initial step of deparaffinization and rehydration was performed by successively immersing skin sections in xylene (2 × 5 min), 100% (v/v) ethanol (2 × 5 min), 95%, 70% and 50% (v/v) ethanol (1 × 3 min each), followed by a 5‐min wash in PBS. An antigen retrieval step was performed to expose the antigenic sites that may have been masked by formalin fixation. The skin and/or aorta sections were incubated with sodium citrate buffer (pH 6.0), heated in a water bath at 100°C for 20 min, and allowed to cool down for 1 h. Sections were then washed with PBS (3 × 2 min). To block nonspecific antibody binding sites and permeabilize the sample, sections were outlined with a hydrophobic pen and incubated with a blocking solution 3% BSA/10% goat serum/0.1% triton X‐100/PBS for 1 h at room temperature. Primary antibodies used were mouse anti‐Progerin (1:250; Sigma), rabbit anti‐Ki‐67 (1:250; Abcam), rabbit anti‐KRT1 (1:1000; BioLegend) and mouse anti‐alpha‐smooth muscle actin Cy3 (1:250; Sigma), diluted in the blocking solution, overnight at 4°C. Antibody–antigen complexes were detected using secondary antibodies Alexa Fluor 568 conjugated goat anti‐rabbit or goat anti‐mouse IgG for 2 h at room temperature. Nuclei counterstaining was performed with Hoechst 33342 (2 μg/mL; Invitrogen Molecular Probes) during secondary antibody incubation. Lastly, sections were rinsed three times with PBS and the coverslips were mounted on glass slides with Mowiol® 4–88 (Sigma) mounting medium. Images were acquired using an Axio Observer Z1 microsocpe (Carl Zeiss) using a Plan‐Apochromat 20×/0.8 M27 objective. Images were analyzed with FIJI (Fiji is Just ImageJ) Software or QuPath Software. The analysis was performed by a researcher who was unaware of the experimental groups.

### 4.11 Statistical analysis

The results are expressed as mean ± SEM. Statistical analyses were conducted using one‐way ANOVA followed by Tukey's multiple comparison test or unpaired Student's *t* test, depending on the number of experimental groups in each experimental condition. Prism 8.4.2 (GraphPad Software) was used for the statistical analysis. The statistical parameters can be found in the figure legends.

## AUTHOR CONTRIBUTIONS

CAA, CC, and MF‐M contributed to the study conception and design. MF‐M, AC, ACF, AL, MB, SC‐S, RA, LC, VL, ACR, and CAA performed experiments, collected, and analyzed data. CL‐O, XN, and LPA provided critical input into data interpretation. MF‐M and AC wrote the original manuscript draft. CAA and CC reviewed and edited the manuscript. All authors read and gave input to the manuscript. CAA and CC were responsible for supervision and funding acquisition.

## CONFLICT OF INTEREST STATEMENT

None declared.

## Supporting information


**Data S1**: Click here for additional data file.

## Data Availability

Data sharing not applicable to this article as no datasets were generated or analyzed during the current study.
